# Cell Tracking for Organoids: Lessons From Developmental Biology

**DOI:** 10.3389/fcell.2021.675013

**Published:** 2021-06-03

**Authors:** Max A. Betjes, Xuan Zheng, Rutger N. U. Kok, Jeroen S. van Zon, Sander J. Tans

**Affiliations:** ^1^AMOLF, Amsterdam, Netherlands; ^2^Bionanoscience Department, Kavli Institute of Nanoscience Delft, Delft University of Technology, Delft, Netherlands

**Keywords:** organoids, cell tracking, lineages, fluorescent markers, cellular dynamics

## Abstract

Organoids have emerged as powerful model systems to study organ development and regeneration at the cellular level. Recently developed microscopy techniques that track individual cells through space and time hold great promise to elucidate the organizational principles of organs and organoids. Applied extensively in the past decade to embryo development and 2D cell cultures, cell tracking can reveal the cellular lineage trees, proliferation rates, and their spatial distributions, while fluorescent markers indicate differentiation events and other cellular processes. Here, we review a number of recent studies that exemplify the power of this approach, and illustrate its potential to organoid research. We will discuss promising future routes, and the key technical challenges that need to be overcome to apply cell tracking techniques to organoid biology.

## Introduction

While the development and maintenance of organs is one of the most fundamental problems in biology, our understanding of it is far from complete. A hallmark of this process is the differentiation of cells in time, in terms of proliferative potential and cell type, with individual cells giving rise to complex lineages that organize in space to shape tissues and organs. Thus far, these differentiation dynamics have often been studied using the *lineage tracing* method (Baron and van Oudenaarden, 2019; McKenna and Gagnon, 2019; Wagner and Klein, 2020). Here, cells are labeled with a heritable marker such as fluorescent genes or a genetic barcode, for instance using Cre-Lox recombination (Snippert et al., 2010; Pei et al., 2017) or lentiviral transduction (Weber et al., 2011; Mohme et al., 2017; Weinreb et al., 2020). This label can be detected in progeny after a certain period by fluorescence microscopy or single-cell sequencing, and hence, allows inference of genealogical relations between cells.

**FIGURE 1:**
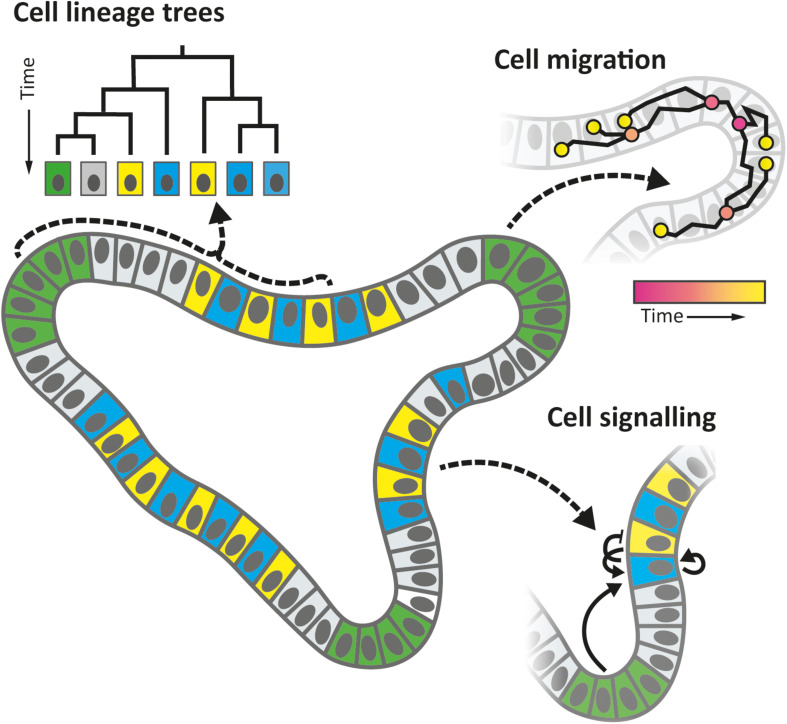
Organoid properties that can be studied by “cell tracking,” a technique in which (nearly) all cells are followed in time over multiple generations, using 3D time-lapse microscopy and automated image analysis, along with a host of fluorescence detection methods. Tracked cell positions allow one to reconstruct lineage trees, follow migration, growth, and division dynamics, while fluorescence reporters may be used to study differentiation events, signaling pathways, and metabolic states, which are key to understand cellular decision-making during development.

However, *lineage tracing* does not yield complete lineage trees nor provide information on the temporal dynamics of cells, such as their movements, growth rates, transient signaling, and timing of differentiation events (Figure 1), which limits progress on many important questions. For instance, it remains largely unclear when and where cell fates are actually set, whether differentiation is either a consequence or a cause of spatial organization, how size and shape homeostasis is achieved, or how lineage dynamics are remodeled upon injury or disease. We also know little about the possible interplay with cellular metabolism, and the plethora of molecular signals from adjacent cells, for instance from the immune system. Elucidating the spatio-temporal dynamics is central to resolve these crucial issues and to elucidate the organizational principles of organ development (Mayr et al., 2019).

A different technique, here referred to as *cell tracking* has the potential to reveal these developmental dynamics. As opposed to *lineage tracing* based on static snapshots, cells are here followed in real time over multiple generations, which can thus provide temporal dynamics, complete lineage trees, as well as spatial organization and cellular movements. Furthermore, *cell tracking* can readily be combined with the large spectrum of microscopy techniques that have been developed to study cell biology. For instance, the expression of fluorescent proteins directly identify differentiation events, cell-cycle progression, cytoskeletal structures, the dynamics of key molecular signals like Wnt or Notch, while FRET sensors may detect more rapidly changing signals such as calcium and metabolites (Lindenburg and Merkx, 2014; Artegiani et al., 2020; van de Moosdijk et al., 2020).

Cell tracking has been applied extensively to study the early development of embryos, at increasing levels of sophistication (Sulston et al., 1983; Ulman et al., 2017; Pennisi, 2018), but poses challenges to the study of organs, given the challenges of time-lapse microscopy deep within tissues at later stages of development, even as intra-vital imaging is possible at lower resolution and throughput (Ritsma et al., 2014). Yet, in recent years organoids have emerged as a model system for studying development and disease at the cellular level, including patient derived systems, which are ideally suited for this approach. Organoids are self-organizing cellular assemblies, which are grown *in vitro* and recapitulate organ structure and functionality to a striking degree (Clevers, 2016; Sidhaye and Knoblich, 2021). Due to their *in vitro* nature, the growth and maintenance of organoid tissue can be observed directly by time-lapse microscopy (Rios and Clevers, 2018). Yet, cell tracking approaches have only scarcely been applied to organoids thus far.

The aim of this review is to discuss the potential of cell tracking approaches for organoid science, as well as its technical challenges. We will do so by focusing on developmental systems ranging from 2D cell cultures to developing embryos, which have been studied extensively by cell tracking methods, and illustrate the scientific questions that it can uniquely address.

## Automated Cell Tracking

Automated tracking of individual cells in time has become a powerful approach to study cellular dynamics in cell lines and embryos (Svensson et al., 2018). Pioneering examples include tracking of embryos of *Caenorhabditis elegans*, fruit flies, zebrafish, and mice (Bao et al., 2006; Amat et al., 2014; Schiegg et al., 2015). In a recent paper, 10^4^ cells were tracked in growing mouse embryos over 48 h, during gastrulation and early organogenesis (McDole et al., 2018). The authors imaged cell nuclei using adaptive multi-view light sheet imaging. Cells were tracked using a Gaussian mixture model, with the center of each nucleus determined by fitting their fluorescent signal to a 3D Gaussian function. By assigning cells present at the end of the experiment to different tissues, e.g., the heart field or the neural tube, based on anatomical features, and by following the tracks of these cells backward in time, it was possible to reconstruct how these cells flowed out of the primitive streak and assembled into tissues. This revealed that the both the timing and position of cells as they left the primitive streak was key to determining their cell fate. Moreover, by tracking cell divisions in time, the authors showed that the orientation of cell divisions changes several times during neural tube closure, with important impact on tissue morphology.

Despite the advance in analyzing mouse embryogenesis, the McDole study also underscores the formidable technical challenges that remain when studying development, including in organoids. While the tracking error rate was low enough to reconstruct the general flow of cells, it was too high to automatically reconstruct lineages in most parts of the embryo. This is because a single erroneous switch in cell identity can corrupt large parts of the lineage tree. Instead, the authors used a mosaic Cre/loxP reporter to sparsely label small subsets of cells. This strongly reduced cell identity mistakes, by increasing the spacing between tracked cells, but severely reduced the lineage information that was captured. While such analysis of cell flow coupled to fate is useful for many applications, acquiring more exhaustive lineage information is of particular importance for organoids, for instance to identify the rare differentiation events and correlations between them, or to reveal spatial interactions on short length scales, including those between neighboring cells that originate from cell–cell signaling.

Following each cell without error requires both fine-tuned image analysis algorithms to accurately identify all nuclei and their positions, as well as a careful balance between limiting phototoxicity and increasing temporal and spatial imaging resolution. Similar to the embryo systems discussed above, organoids have extended 3D structures that lead to out-of-focus light and scattering, resulting in decreased resolution. In addition, cell nuclei tend to be more closely packed compared to early embryos and, particularly in epithelia, nuclei move rapidly along the apicobasal axis during division (McKinley et al., 2018). These properties require comparatively fast imaging (one 3D image every 5–15 min) at high spatial resolution (better than 1 μm/pixel), and generally complicates identification of all nuclei in each frame, for instance using gaussian fitting, and linking them through time without error. Light-sheet imaging may be used (Rios and Clevers, 2018; Serra et al., 2019), which can limit resulting phototoxicity. This technique has also been used to study the flow of embryonic renal cells in kidney organoids during kidney rudiment re-aggregation (Held et al., 2018). However, more broadly available confocal imaging is often sufficient for organoid time-lapse imaging studies.

Apart from imaging, the dense 3D tissues found in organoids also pose challenges for nuclei identification using established image analysis approaches, such as Gaussian mixture models. An important recent advance in this regard is the use of neural networks and machine learning. This approach, which is based on a training procedure that uses manually analyzed datasets to learn to identify nuclei, was shown to improve performance in non-organoid systems with closely packed cells (Ulman et al., 2017; Xing et al., 2018). Another issue is that cell tracking software can be difficult to use for non-experts (Meijering et al., 2016). Cell trackers often need to be reprogrammed, reconfigured, or retrained in the case of a neural network approach, upon changes in the system studied, imaging parameters, or fluorescent reporters, although algorithms that work for a wider range of microscopy images are developed (Stringer et al., 2021). Finally, an important practical problem is that software packages are not always well suited to correct for the tracking errors they invariably generate. This feature is less important when studying properties such as cell flow, but is important for lineage analysis in organoids, where differentiation events may be strongly influenced by stochasticity or neighbor interactions.

As a consequence, manual cell tracking approaches are still used, even for systems with hundreds of cells (Wolff et al., 2018). A promising new direction for organoid systems is to combine automated tracking based on neural networks with manual error correction steps. Such an approach was used to reconstruct cell lineages by tracking 50 cells during embryonic brain regionalization in brain organoids (Giladi et al., 2020). Key to scaling up such a hybrid approach from a limited number of lineages to entire organoids is to incorporate algorithms that automatically identify possible errors and allow for efficient manual correction of these errors. Recently, we developed such a hybrid approach to perform lineage tracking for whole intestinal organoids (Kok et al., 2020).

In the future, we expect that automatic cell tracking approaches will continue to improve, driven in part by advances in machine learning methods (Svensson et al., 2018). Currently cell tracking studies focus primarily on cellular movement and divisions. With automatic cell tracking becoming more accessible, a range of new applications will open up in organoid research, including the study of cellular differentiation, tissue renewal, shape and symmetry changes, and may involve simultaneous measurements of key regulatory and metabolic signals (Kiviet et al., 2014; Rodríguez-Colman et al., 2017).

## Cell Tracking Combined With Fluorescent Markers

The tracking of cells and their corresponding lineage trees by itself is often not enough to understand how developmental decisions are made. The results of these decisions, the cell fates, are invisible in most situations – with the exception of well-characterized systems where the cell type can be deduced from its spatiotemporal position and anatomical features, like the mouse embryo discussed in the previous section. By combining fluorescent markers that report on cell type with image-based cell tracking, it is possible to monitor a cell’s identity, position and lineage dynamics concurrently, and hence, study where and when cell fate decisions are made.

A recent study (Viader-Llargués et al., 2018) showcases the power of this combination. This work focused on tissue regeneration upon damage of the neuromast, a small sensory organ, in zebrafish. An elegant combination of cytosolic and nuclear markers allowed imaging and identification of all three major cell types in the neuromast using only two colors. These fluorescence markers made it possible to selectively photo-ablate different parts of the organ, and to subsequently study the regenerative potential of the different cell types. By manually tracking lineages it was found that multiple cell types have regenerative potential, but only one type has the potential to regenerate all three major cell types in the neuromast.

The authors then aimed to understand how these cell fate decisions are regulated to faithfully regenerate the organ from a single cell type. To analyze the large amounts of data generated during live imaging, feature lists were compiled for every tracked cell, including both intrinsic (e.g., the time since birth) and extrinsic information (absolute position, relative position to other cell types, and polar orientation). Using a machine learning technique called “random forest” to predict cell fate decisions, spatial features, like the position of cells relative to the organ center during division, were shown to be highly predictive of the cell fates that their progeny will take on. Intrinsic features were uninformative, suggesting that in this system, cell fates are not determined by (prior) cellular heterogeneity but by the cells plastically responding to their environment. This influence of position on cell fate would have been difficult to determine without the combination of live fluorescence markers and image-based lineage tracking.

For organoids, this approach is increasingly feasible, especially given recent progress in CRISPR based techniques that allow fluorescent reporters to be directly incorporated in organoid lines (Artegiani et al., 2020). An organoid model for breast cancer has been used to study why some cells carrying an oncogenic mutation become highly proliferative while others do not (Alladin et al., 2020). Tracked lineages and a fluorescent reporter for the mutation indicated that the local density of mutated cells was the most predictive feature. Being within a cluster of other mutated cells yielded increased progeny. Again, the combination of spatial and lineage information (in this case the amount of progeny) provided by cell tracking were central to the conclusions.

We note that the breast organoids from Alladin et al. (2020) and the neuromasts studied by Viader-Llargués et al. (2018) are comparatively small systems and contained few cell types. Tracking will be more challenging in larger systems of several hundreds of cells, while spectral overlap limits the number of fluorescent labels, and hence, the ability to distinguish all cell types of interest. Besides reporting for cell type, fluorescent proteins can also quantify cellular processes in organoids, such as chromosome and tubulin dynamics during cell division (McKinley et al., 2018; Bolhaqueiro et al., 2019; Artegiani et al., 2020). Even metabolic processes like oxygenation can be followed using fluorescence sensors (Okkelman et al., 2017). Also promising are fluorescence reporters for the signaling pathways that regulate developmental decisions. Often, these reporters can be fused either directly to a downstream target of the pathway or placed under control of a target gene promotor. Short-lived fluorescent proteins might be required to detect rapid pathway activity dynamics (Doupe and Perrimon, 2014). Indeed, newly developed Wnt and Notch reporters have shown notable dynamics, which in turn impact differentiation (Delaune et al., 2012; Sonnen et al., 2018; Massey et al., 2019; Rosenbloom et al., 2020). These functional read-outs can be readily combined with lineage tracking to quantitatively study correlations with cellular organization and differentiation.

## Cell Tracking Combined With End-Point Measurements

As discussed in the previous section, signaling dynamics and changes in cell state during development can in principle be monitored directly using fluorescent markers. However, the spectral overlap of fluorescent proteins and the time investments associated with the required genetic engineering limits the number of colors that can be imaged simultaneously to at most 2–3. This severely limits the number of cell types or genes that can be tracked in one experiment.

An approach that may help to circumvent this limitation was used to study 2D cultures of mouse embryonic stem cells (Hormoz et al., 2016). After tracking the cells over time, these same cells were subsequently studied using three-color single molecule RNA-fluorescence *in situ* hybridization (RNA-FISH). These data quantified the expression of three genes that mark various differentiation stages. Each cell was classified as high or low for each gene, resulting in eight possible cell states. These states were correlated with the lineage history of each cell using an analytical approach called Kin Correlation Analysis (KCA). This method infers the cell-state transition rates during time-lapse imaging by analyzing state-correlations between relative cells, such as sisters and cousins. Reversible transitions occurred only between adjacent cell states, in a linear chain of cell states from pluripotent to more differentiated. Overall, these experiments show how developmental dynamics can be inferred by combining dynamic cell tracking with static end-point measurements.

One advantage of such end-point measurements is their scalability. In order to find genes important to *Escherichia coli* cell cycle control, cells from a library of 235 CRISPR interference (CRISPRi) perturbations were tracked for days jointly in a single experiment, while characterizing phenotypes such as chromosome replication forks, cell size, and growth rates. Afterward, 10 sequential rounds of FISH labeling were performed to identify the underlying CRISPR perturbation in each tracked lineage. Key replication initiation regulators could hence be identified, and yielded new replication initiation control models (Camsund et al., 2020). Measurements of gene expression in single fibroblast cells and in brain tissue have been performed using a sequential staining method termed multiplexed error-robust FISH (MERFISH) (Chen et al., 2015; Moffitt et al., 2016a,b). Applying smFISH to organoids is challenging, due to limited penetration of probes and high background fluorescence, and often requires cryosectioning, which is non-trivial to implement (Grün et al., 2015). However, a protocol for 3D smFISH in whole-mount colon organoids, that relied on reducing the background fluorescence of Matrigel, was described recently and may be applicable to other organoid systems (Omerzu et al., 2019).

This approach may be extended to other end-point analysis techniques such as immunostaining. Immunostaining has been often used to study cell type and differential expression statically, without a combination with cell tracking, for instance recently in neural tube development (Fannon et al., 2021). Multiplexed immunofluorescence imaging techniques allows for more than ten sequential rounds of antibody staining in a tissue (Lin et al., 2018). Multiple rounds of immunostaining has been used in intestinal organoids (Serra et al., 2019), though not in combination with cell tracking. An exciting idea is performing single-cell RNA-sequencing (scRNA-seq) as end-point analysis after cell tracking, as it would allow one to correlate the lineage tree to genome-wide changes in gene expression. However, as cells may then have to be removed from their tracked location to perform sequencing, which may be partially achieved by sorting fluorescently labeled cells or microaspiration, challenges remain in linking these different data sets. Alternatively, cell tracking could be followed by the recently developed spatial transcriptomics technique, which allows for RNA-sequencing while keeping the information of relative cell positions, though current techniques use sectioning and do not provide 3D information (Ståhl et al., 2016; Lein et al., 2017; Rodriques et al., 2019). Overall, combining lineage tracking with static end-point measurements in organoids provides a promising approach to study spatio-temporal organization in developing organoids.

## Discussion

In this review we have highlighted a number of recent studies that show the power of cell tracking approaches and how they can be applied to organoid model systems. They first of all underscore a general reality: the more we follow biological processes in time, the more crucial dynamics we uncover. This is perhaps particularly true for developmental processes, where organization in time is the core of the problem. They also illustrate how key technologies are now converging. On the one hand, advances in 3D microscopy and image analysis algorithms provide increasingly detailed views of cellular dynamics. On the other hand, rapid progress in genetic engineering and single-cell sequencing yield ever more information on key regulators and markers of cell identity. With adjustments, these techniques are highly suited for application to the biology and biophysics of organoids, where study of temporal dynamics is still in its infancy. Together, these developments now provide an exciting opportunity to understand the underlying principles by which organs and organoids are organized in space and time.

While the studies reviewed here show that the main technologies are available, numerous improvements and extensions can be envisioned. At a practical level, more reliable automated tracking of cell movement and division would greatly expand the general use and throughput of cell tracking approaches. The development of new fluorescent reporters and sensors promises far more detailed observations of regulatory and metabolic pathways than is possible currently. Approaches that can link cell tracking to more expansive cell expression measurements, including multiplexed immunostaining, smFISH, and single-cell sequencing, have the potential to unlock a next level of understanding down to the molecular scale.

A general challenge will be to analyze the resulting lineage tracking data sets, given their complex, multi-faceted nature that combines space, time, lineage, and internal states. How can insight into simple organizing principles be inferred from such data sets? One approach could be to use machine learning techniques to identify the most relevant features informing cell fate decisions (Ståhl et al., 2016; Viader-Llargués et al., 2018; Alladin et al., 2020). In addition, one may exploit dimensionality reduction techniques similar to ones used in the single cell sequencing and flow cytometry fields, which deal with similar highly dimensional single-cell resolved data, as they could identify hidden structures in the data. For instance, currently expressed cellular fate could be determined by past cell–cell contacts, cellular location, orientation, and molecular signals, while conversely, cellular migration speeds and spatial patterns may depend on cell type, age, and genealogical relations. Reduction techniques are already being used to analyze high throughput microscopy (Caicedo et al., 2017; Czech et al., 2019) and recently have been used in the analysis of time-resolved imaging of whole organoids (Serra et al., 2019) and single cells in 2D culture (Rennerfeldt et al., 2019). Beyond the single cell level, recently developed methods allow statistical analysis of lineage trees shapes (Stadler et al., 2018; Yuan et al., 2020) and simultaneously measured phenotypic signals (Kiviet et al., 2014; Feigelman et al., 2016; Skylaki et al., 2016; Stadler et al., 2018; Hicks et al., 2019). In this way key differences between lineages may be identified and correlated with developmental decisions. However, it is likely that new analysis methods and the introduction of bottom-up mechanistic models are needed to make full use of the incredible richness of information that these new technologies can provide, and allow one to move from a descriptive to a predictive understanding of organ biology.

## Author Contributions

The work was written and edited by all authors. All authors listed have made a substantial, direct and intellectual contribution to the work, and approved it for publication.

## Conflict of Interest

The authors declare that the research was conducted in the absence of any commercial or financial relationships that could be construed as a potential conflict of interest.
